# Unravelling the role of mitochondrial DNA in hybrid incompatibility within species of the *Anopheles gambiae* complex

**DOI:** 10.1038/s41598-024-80887-0

**Published:** 2024-11-27

**Authors:** Antonios Kriezis, Matteo Vitale, Giulia Morselli, Andrea Crisanti, Federica Bernardini

**Affiliations:** Department of Life Sciences, Imperial College London, London, UK

**Keywords:** Hybrid incompatibilities, Genetics, Introgression, Mosquitoes, Anopheles gambiae complex, Entomology, Disease vectors, Mitochondrial DNA, Haldane Rule., Cell biology, Evolution, Genetics

## Abstract

Isolation mechanisms between mosquito species of the *Anopheles gambiae* complex, which includes major malaria vectors, remain poorly understood. In some cases, pre-zygotic barriers have been shown to limit gene flow between species of the complex, leading to a low level of hybridisation in nature. Post-zygotic mechanisms manifest with F_1_ hybrid males fully sterile and F_1_ hybrid females with reduced fertility. Genetic approaches combined with DNA sequencing techniques have highlighted the involvement of genomic regions in hybrid incompatibility with a predominant role of the X chromosome. In addition, differences in the phenotype of F_1_ hybrid males have been identified depending on the directionality of the parental cross used to generate them. All these studies have focused on the interaction of nuclear DNA elements in hybrid individuals. Given the role that mitochondrial DNA plays in genetic incompatibilities within other organisms and its unique inheritance pattern, commonly maternal, we conducted a genetic study that relied on the introgression of mitochondrial DNA between *Anopheles gambiae* and *Anopheles arabiensis*. The findings indicate that the mitochondrial switch does not appear to restore the fertility of F_1_ hybrid males, suggesting that mitochondrial DNA may not play a role in hybrid incompatibilities in these Anopheles species.

## Introduction

Malaria continues to pose a substantial global health challenge, with an annual infection rate exceeding 200 million individuals and an estimated half a million fatalities reported each year. The African continent continues to experience the most significant impact of this burden^1^.

Major vectors of malaria in Africa belong to the *Anopheles gambiae sensu lato* (s.l.) (Diptera: Culicidae) species complex, which includes nine morphologically indistinguishable species of mosquitoes that significantly differ in their vectorial capacity^2,3^.

Prezygotic isolating mechanisms have been observed in some of these species, resulting in assortative mating. These factors, which are still poorly understood, include temporal, ecological, behavioural, and mechanical isolation^4–12^. In addition, post-zygotic isolation mechanisms, in the form of interspecific hybrid incompatibilities (HIs), have been identified and act to restrict gene flow: interspecific crosses between members of the complex produce sterile F_1_ hybrid males^13–17^. A notable exception to this pattern can be found in crosses between *An. gambiae* and *An. coluzzii*, the youngest species pair to diverge within the complex^18^. In accordance with Haldane’s rule, while F_1_ hybrid males express full sterility, homogametic F_1_ female hybrids between species of the complex are fertile, conditional on viability, providing still poorly understood opportunities for gene flow^3,18–23^. Nevertheless, both entomological and molecular methods suggest that hybridisation rates in African populations of Anopheles mosquitoes are relatively low^9,24–29^.

One of the most prominent models of speciation is based on the concept that mutations can occur and accumulate within the genomes of isolated populations. These mutations can confer an adaptive advantage or be neutral within these populations; however, if hybridisation between previously isolated populations occurs, the presence of these alleles in a hybrid genetic background could give rise to deleterious effects such as unviability, sterility or, more generally, fitness costs. The detrimental effects due to these genetic variants are known as Dobzhansky-Muller or Bateson-Dobzhansky-Muller incompatibilities (BDMIs), and examples of their significance in HIs and reproductive isolation are well documented^30–37^.

Several studies have attempted to elucidate the genetic mechanisms involved in HIs within the *Anopheles gambiae* complex. Introgression experiments have ruled out the role of the Y chromosome in HIs for *An. gambiae* and *An. arabiensis* hybrids^15^. On the other hand, in hybrids between these species, as well as between *An. coluzzii* and *An. quadriannulatus*, a prominent role of the X chromosome, has been highlighted^38^. In addition, autosomal regions have been identified as having an effect on hybrid fertility to various extents^14,39,40^.

A common feature of all genetic elements so far studied is that they are determined by nuclear DNA. However, several examples of reproductive barriers in eukaryotic organisms involve interactions between nuclear and mitochondrial genes^41–43^.

Mitochondria are membrane-bound cell organelles that, in eukaryotic cells, supply chemical energy for biological processes. These organelles carry a genome (mitogenome) that is inherited independently of the nucleus, and they are typically small and characterised by low levels of recombination. Interactions between mitochondrial and nuclear genes are crucial for proper cell function, and misregulation can cause severe pathological effects^44^. Importantly, mitochondrial genomes are uniparentally, usually maternally, inherited^45^. These unique features indicated that mitogenome-nuclear DNA should be considered as a candidate for the manifestation of interspecific BDMIs^46^. Furthermore, due to the maternal inheritance pattern, mutations in the mitochondrial genome are only subjected to natural selection in females. This means harmful mutations that affect males can persist in populations if beneficial or neutral in females. In males, selective pressure would favour the evolution of nuclear variants that restore mitonuclear interactions. This phenomenon, known as the Mother’s Curse^47–49^, could contribute to BDMIs.

Interestingly, Liang & Sharakhov (2019) and Liang et al., (2021) recently investigated F_1_ hybrid male sterility between *An. merus* and *An. gambiae* or *An. coluzzii*. In accordance with previous observations, the authors showed that while F_1_ hybrid males resulting from either reciprocal parental cross were fully sterile, an asymmetrical sex bias was observed in the offspring depending on the directionality of the parental cross^50,51^. In addition, an asymmetrical pattern also manifested in the cytological mechanisms underlying the sterility of F_1_ hybrid males (Darwin’s corollary)^16,17^. Given that autosomes are shared between male and female mosquitoes, the phenotype difference relative to the genetic crosses’ directionality could be attributed to the sex chromosomes. Alternatively, the mitogenome could be involved in these mechanisms.

Due to its high mutation rate (2 to 6 times faster than nuclear DNA in non-vertebrates), mitochondrial DNA has often been studied to investigate speciation and evolution^52,53^. In 2019, Hanemaaijer and colleagues identified 783 single nucleotide polymorphisms (SNPs) in the mitogenome of field-collected *An. arabiensis*, *An. gambiae* and *An. coluzzii*^54^. None of these SNPs was unique to either species, suggesting that there is no divergent selection in the mitogenome among these species of the *Anopheles gambiae* complex, possibly due to hybridisation events. These results suggest that the mitogenome may not play a role in HIs^54^.

However, empirical studies that follow a traditional route of investigation that account for the complexity of biological factors have, to our knowledge, never been performed to investigate cytonuclear interactions in mosquitoes.

This study used a genetic cross scheme to introduce the mitogenome deriving from *An. arabiensis* into an *An. gambiae* genetic background and the mitogenome deriving from *An. gambiae* into an *An. arabiensis* genetic background. These genetic makeups allowed generations of F_1_ hybrids with a switch in their mitochondrial DNA composition and the possibility of exploring the role of mitochondria in HIs between these mosquito species.

## Results

### Cytology of reproductive organs in F_1_ hybrid males of *an. Gambiae* and *An. Arabiensis*

We employed two laboratory strains to investigate hybrid incompatibility in Anopheles mosquitoes: *An. gambiae* G3 (referred to as *An. gambiae* hereafter) is a hybrid strain of *An. gambiae* and *An. coluzzii*, and *An. arabiensis* Dongola (referred to as *An. arabiensis* hereafter).

We and others have previously shown that, in accordance with Haldane’s rule, F_1_ hybrid males generated between these strains displayed full sterility^14,15,40,55^.

**Fig. 1:**
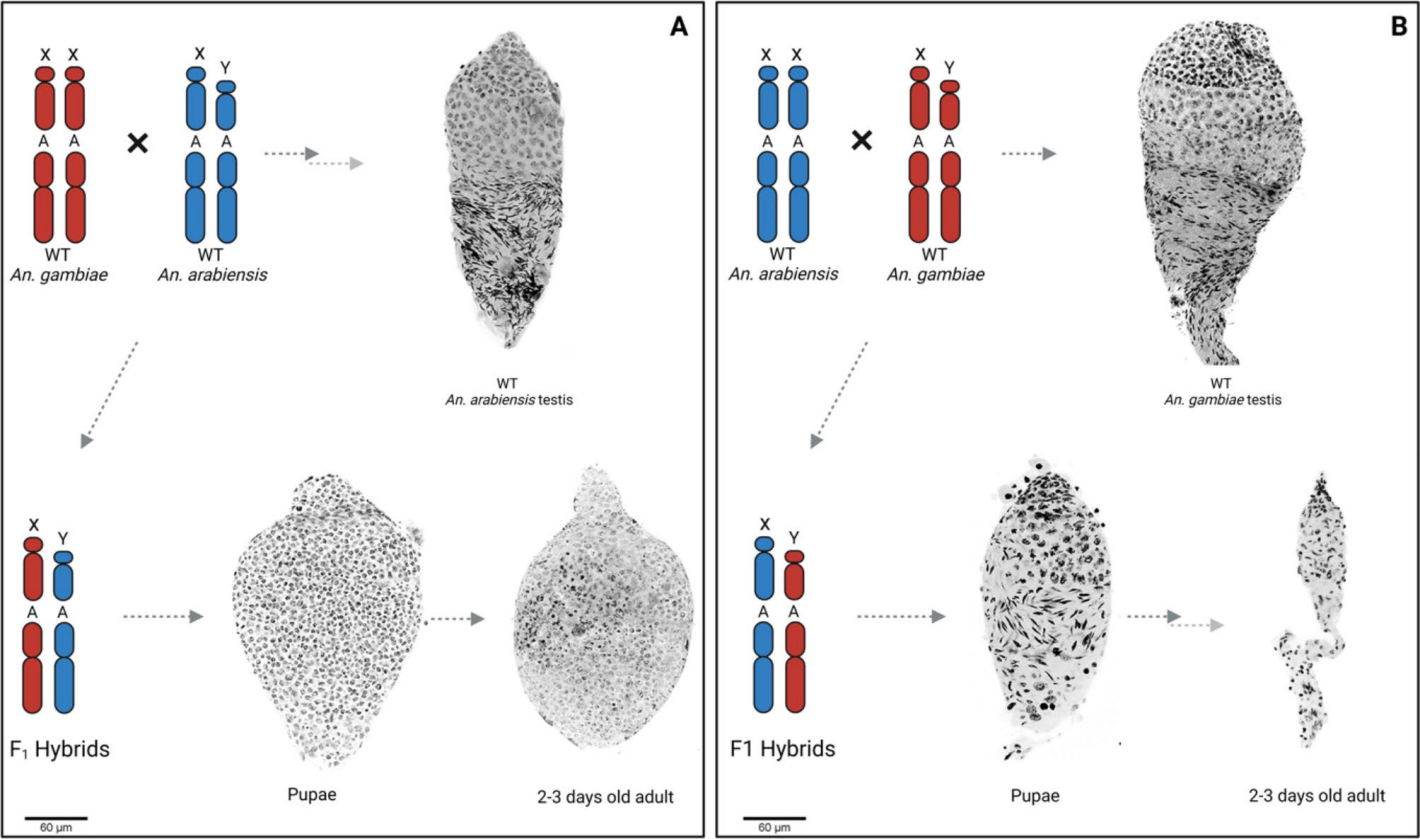
Whole mount DAPI staining showing the cytological aspect of testes associated with reciprocal crosses between *An. gambiae* and *An. arabiensis*. Representative chromosomes are labelled with X (X chromosome), Y (Y chromosome) and A (autosome). Red indicates *An. gambiae* DNA, blue indicates *An. arabiensis* DNA. (**A**) WT *An. arabiensis* testis dissected from adult males show different stages of spermatogenesis. Large spermatocyte nuclei can be detected in the upper part of the testis. Mature, arrow-shaped spermatozoa are present in the sperm reservoir in the lower part of the organ. F_1_ hybrid males resulting from a cross of *An. gambiae* females to *An. arabiensis* males have ‘marbled’ testes where the characteristic spermatogenesis stages and mature spermatozoa are absent. (**B**) Testes dissected from WT *An. gambiae* adult males match the cytological phenotype described for WT *An. arabiensis* males. F_1_ hybrid males were generated from a cross of *An. arabiensis* females to *An. gambiae* males. At the pupal stage, cells at different stages of spermatogenesis can be seen, however, sperm cells show chromatin condensation defects and are larger in size compared to sperm in WT individuals. Following the transition from pupa to adult mosquito, testes undergo progressive degeneration, their size is significantly reduced (‘atrophic’) and the different stages of spermiogenesis are poorly defined.

Interestingly, the severity of the morphological anomalies of the reproductive organs underlying this sterility varied according to the directionality of the parental cross. F_1_ hybrid males resulting from a cross of *An. gambiae* females to *An. arabiensis* males showed testes that were similar in shape and size to those of Wild Type (WT) males of either species but appeared to contain mostly large, round, undifferentiated cells. This phenotype was termed ‘marbled’ (Fig. 1A). When testes of this hybrid male cohort were squashed using a glass coverslip, some spermatozoa with partial tails were observed, though none were mature or motile (Figure S1).

Conversely, F_1_ hybrid males resulting from a cross of *An. arabiensis* females and *An. gambiae* males showed much smaller testes than WT males and were largely deprived of cellular components. This phenotype has been termed ‘atrophic’ (Fig. 1B). Notably, testes dissected from this cohort of hybrid males at the pupal stage seemed normal, indicating a progressive organ degeneration between the pupal stage and early adulthood (Fig. 1B). In pupae, DAPI staining revealed the presence of distinct developmental stages of spermatocytes and spermatozoa with chromatin condensation defects; none of these cells were motile (Figures S2, S3).

### Generation of mosquito strains with interspecific mitochondrial and nuclear DNA

**Fig. 2:**
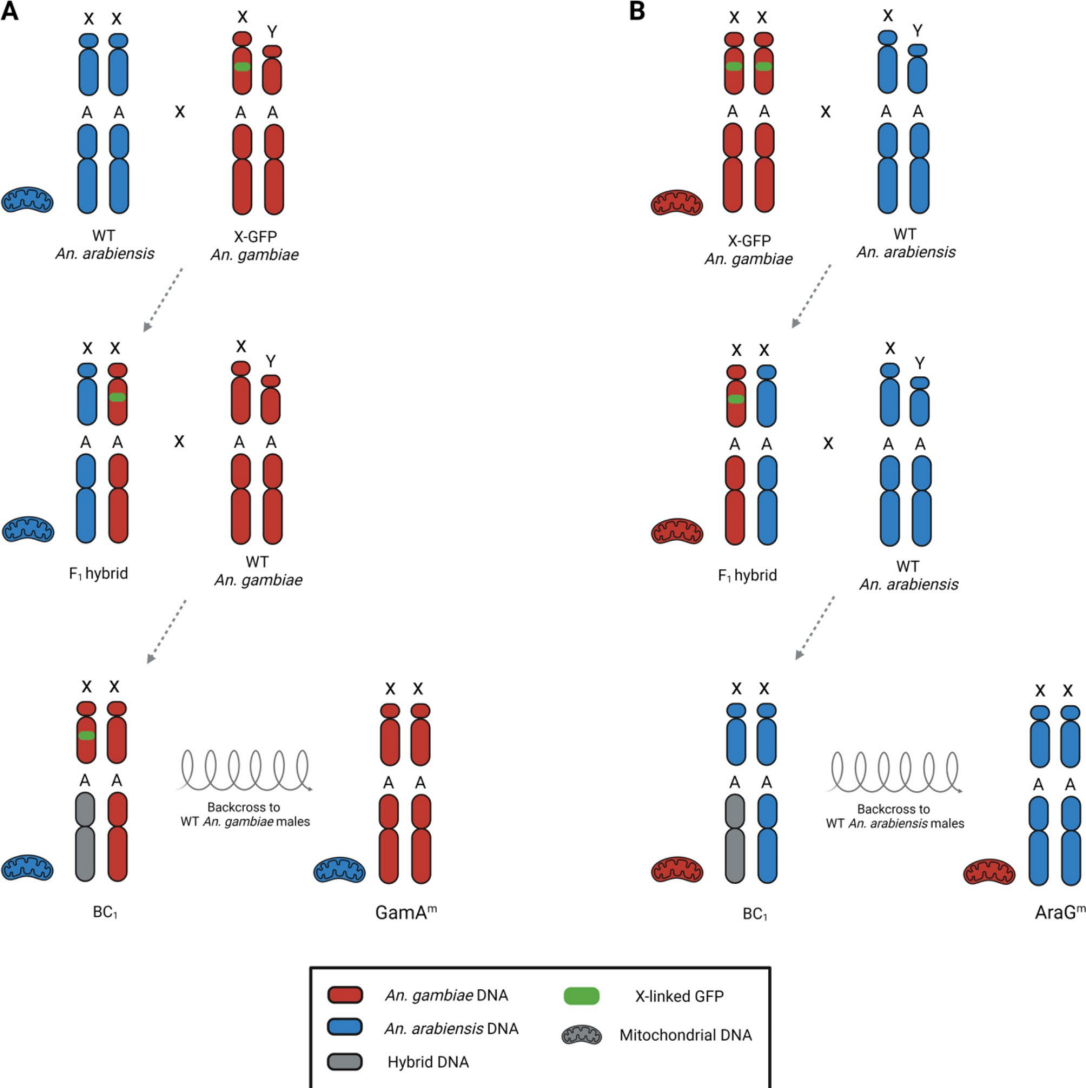
Schematics for the interspecific introgression of mitochondrial DNA. Representative chromosomes are labelled with X (X chromosome), Y (Y chromosome) and A (autosome). Red indicates *An. gambiae* DNA, blue indicates *An. arabiensis* DNA, grey indicates hybrid DNA and green denotes the X-linked GFP transgene. *(****A****)* Introgression of *An. arabiensis* mitochondria into an *An. gambiae* genetic background. WT *An. arabiensis* females were crossed to *An. gambiae* X-GFP males in cage. F_1_ hybrid females were backcrossed to WT *An. gambiae* males, and transgenic BC_1_ hybrid females were selected to continue the introgression by repeated backcrosses to WT *An. gambiae* males. The X-linked GFP transgene is no longer used for the selection of the individuals and is eventually lost. *(****B****)* Introgression of *An. gambiae* mitochondria into an *An. arabiensis* genetic background. X-GFP *An. gambiae* females were crossed to WT *An. arabiensis* males in cage. F_1_ hybrid females were backcrossed to WT *An. arabiensis* males and non-transgenic BC_1_ hybrid females were selected to continue the introgression by repeated backcrosses to WT *An. arabiensis* males.

A scheme of introgressive hybridisation was employed to introduce *An. arabiensis* mitochondria into the genomic background of *An. gambiae* (Fig. 2A).

WT *An. arabiensis* females were crossed *en masse* to transgenic *An. gambiae* males carrying a fluorescent marker on the X chromosome (X-GFP strain). Transgenic F_1_ hybrid females were then backcrossed to WT *An. gambiae* males *en masse* to generate backcross generation 1 (BC_1_) progeny. From this cohort, transgenic females, which had inherited one set of chromosomes from their WT *An. gambiae* fathers and one set of chromosomes that resulted from meiotic recombination in their F_1_ hybrid mothers, were selected.

Recombination rates between interspecific X chromosomes have been shown to be very low in anopheline mosquitoes^14,38–40^. As a result, the presence of the X-linked GFP marker was used to select BC_1_ females that had inherited the *An. gambiae* X chromosome from their F_1_ hybrid mothers, thus eliminating the *An. arabiensis* X chromosome from this introgression (Fig. 2A).

To further decrease the amount of nuclear *An. arabiensis* DNA in the introgressed strain, BC_1_ females were backcrossed to WT *An. gambiae* males, and this backcrossing scheme was employed for over 20 subsequent generations (more than one year). By selecting female progeny for each subsequent backcross, we ensured that the *An. arabiensis* mitochondrial DNA was retained and at generation BC_26_, this strain was expected to contain the *An. arabiensis* mitochondrial DNA within an *An. gambiae* genomic background. We refer to this strain as GamA^m^ (Fig. 2A).

In parallel with this introgression experiment, the same strategy was used to generate a strain called AraG^m^, which carries *An. gambiae* mitochondrial DNA in an *An. arabiensis* genetic background (Fig. 2B).

### Generation of F_1_ hybrids containing heterospecific X-chromosomes and mitochondrial DNA

**Fig. 3:**
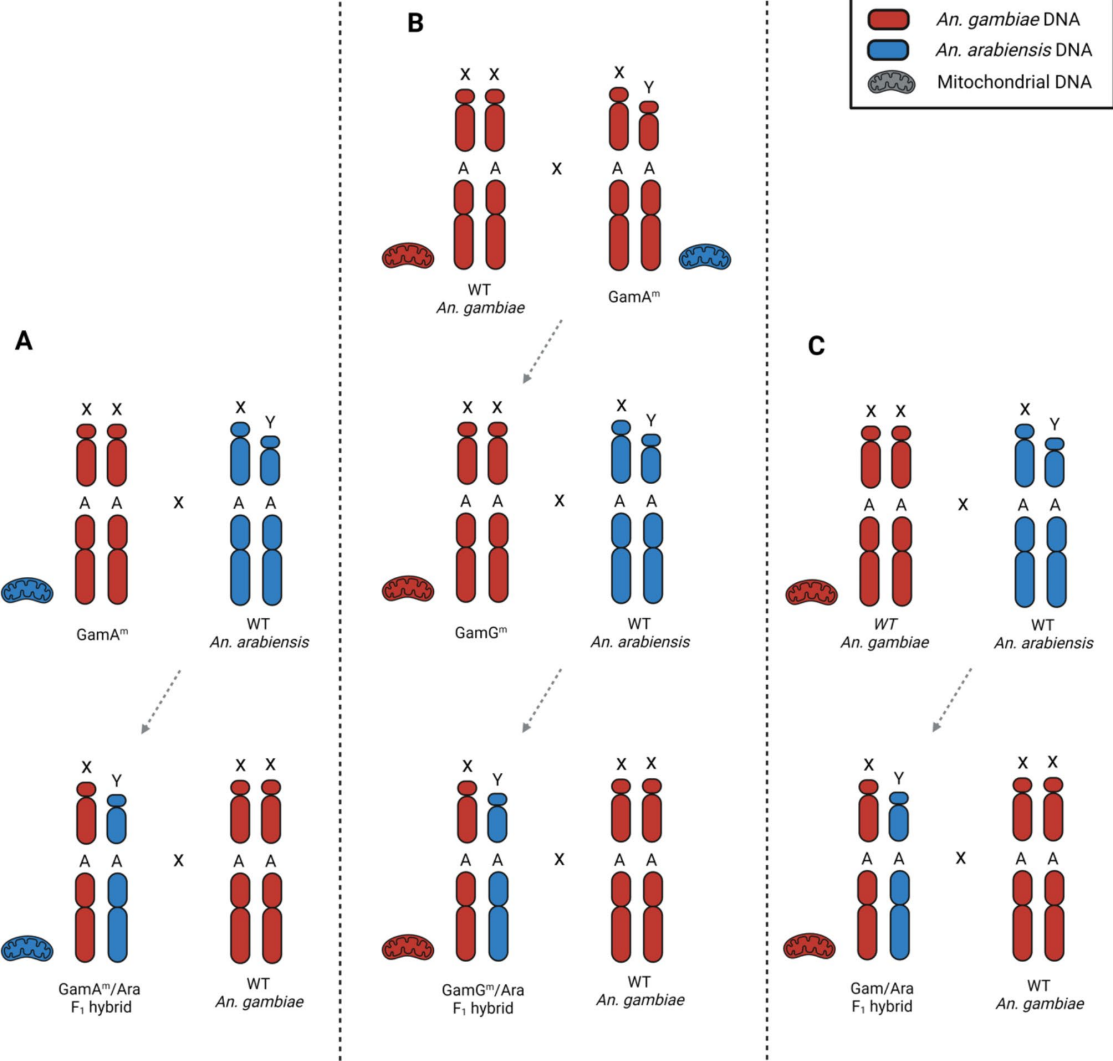
Genetic crosses performed to assess the effect of *An. arabiensis* mitochondria in F_1_ hybrid males containing the *An. gambiae* X chromosome. Representative chromosomes are labelled with X (X chromosome), Y (Y chromosome) and A (autosome). Red indicates *An. gambiae* DNA, blue indicates *An. arabiensis* DNA. (**A**) GamA^m^ females were crossed in cage to WT *An. arabiensis* males to generate GamA^m^/Ara F_1_ hybrid males containing *An. arabiensis* mitochondria and *An. gambiae* X chromosomes. (**B**) GamA^m^ males were crossed to WT *An. gambiae* females in cage to generate GamG^m^ females which had the same genomic species-composition as the GamA^m^ strain but contained *An. gambiae* mitochondria. These females were crossed to WT *An. arabiensis* males in cage to generate GamG^m^/Ara F_1_ hybrid males containing both the mitochondria and the X chromosome from *An. gambiae*. (**C**) As a control, F_1_ hybrid males were generated by crossing WT *An. arabiensis* males to WT *An. gambiae* females in cage. The Gam/Ara F_1_ hybrid males contained a set of autosomes from each species and mitochondria from *An. gambiae*.

**Fig. 4:**
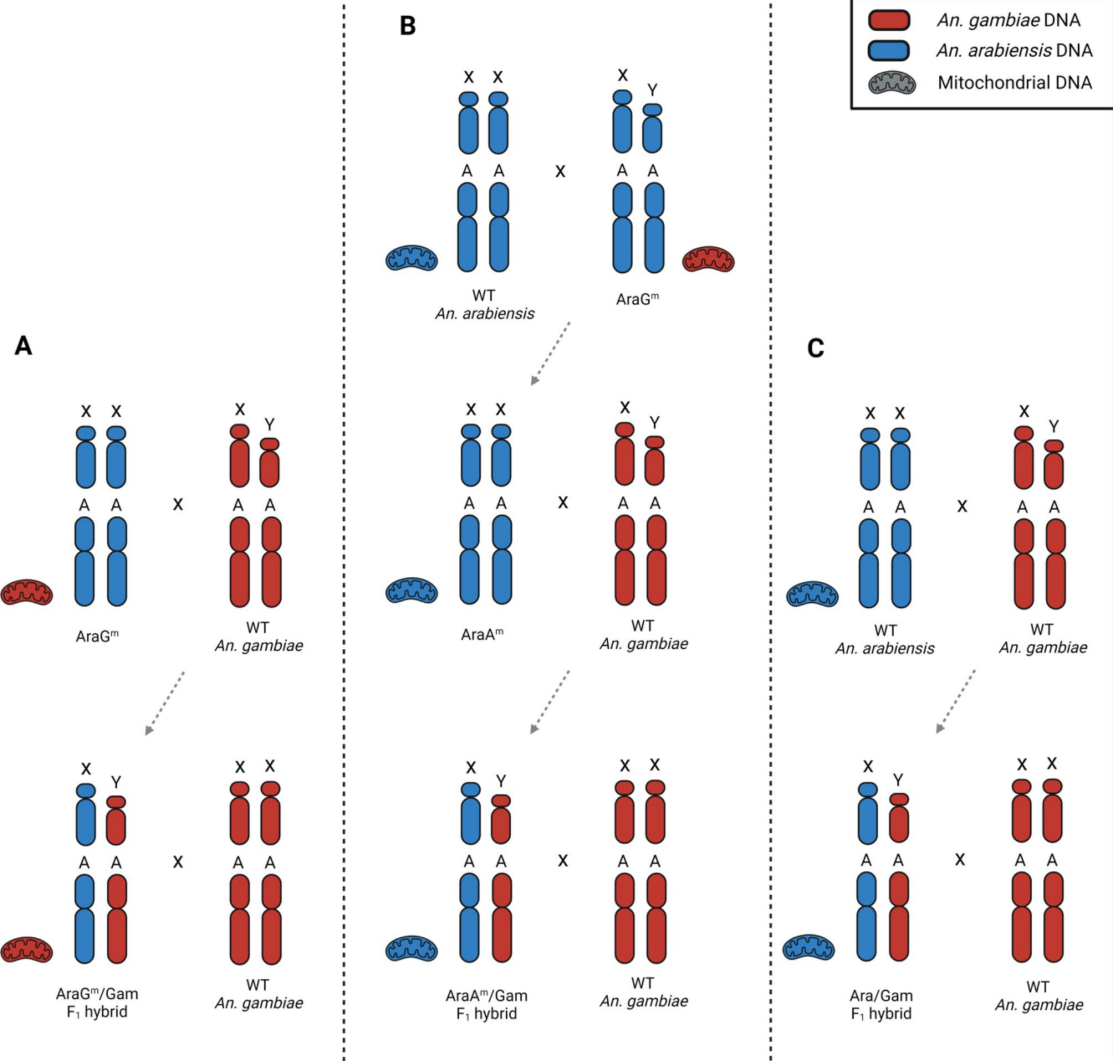
Experimental crosses to assess the effect of *An. gambiae* mitochondria on testes phenotype and fertility in F_1_ hybrid males containing the *An. arabiensis* X chromosome. Representative chromosomes are labelled with X (X chromosome), Y (Y chromosome) and A (autosome). Red indicates *An. gambiae* DNA, blue indicates *An. arabiensis* DNA. (**A**) AraG^m^ females were crossed in cage to WT *An. gambiae* males to generate AraG^m^/Gam F_1_ hybrid males containing *An. gambiae* mitochondria and the *An. arabiensis* X chromosome. (**B**) AraG^m^ males were crossed to WT *An. arabiensis* females in cage to generate AraA^m^ females which had the same genomic species composition as the AraG^m^ strain but contained *An. arabiensis* mitochondria. These females were crossed to WT *An. gambiae* males in cage to generate AraA^m^/Gam F_1_ hybrid males containing both the mitochondria and the X chromosome from *An. arabiensis*. (**C**) As a control, F_1_ hybrid males were generated by crossing WT *An. gambiae* males to WT *An. arabiensis* females in cage. The Ara/Gam F_1_ hybrid males contained a set of autosomes from each species and mitochondria from *An. arabiensis*.

This study aimed to investigate any possible effect of the presence of heterospecific X-chromosomes and mitochondrial DNA in the genomic context of F_1_ hybrid males. Therefore, we designed a scheme of genetic crosses utilising the introgressed strains GamA^m^ and AraG^m^, as well as WT *An. arabiensis* and *An. gambiae.* By using this strategy, we produced hybrid males that inherited a set of chromosomes (autosomes/X chromosome) from one parental species in conjunction with a set of chromosomes (autosomes/Y chromosome) and mitochondrial DNA from the other species (Figs. 3A and 4A).

To ensure that any alteration of the sterility phenotype observed in these hybrid males was the result of the mitochondrial DNA switch rather than incomplete DNA introgression in the genome of GamA^m^ and AraG^m^ strains, we set up genetic crosses that aimed at generating hybrid males into which mitochondrial DNA had been reintroduced to match the species-of-origin of the X chromosomes (Figs. 3B and 4B).

In parallel, we generated F_1_ hybrid males from WT *An. arabiensis* and *An. gambiae* strains to serve as a control (Figs. 3C and 4C).

Hybrid males of the experimental and control groups were crossed *en masse* to WT *An. gambiae* females to assess their fertility. Moreover, testes were dissected and analysed to determine their phenotype.

### Sterility in F_1_ hybrid males is not affected by the presence of heterospecific mitochondria and X chromosomes

Upon mating with hybrid males and blood feeding, WT *An. gambiae* female mosquitoes from the experimental and control cages were allowed to lay eggs. The number of eggs deposited and the hatching rate (number of larvae/number of eggs) were used to assess the fertility of the cohorts of F_1_ hybrid males.

**Fig. 5:**
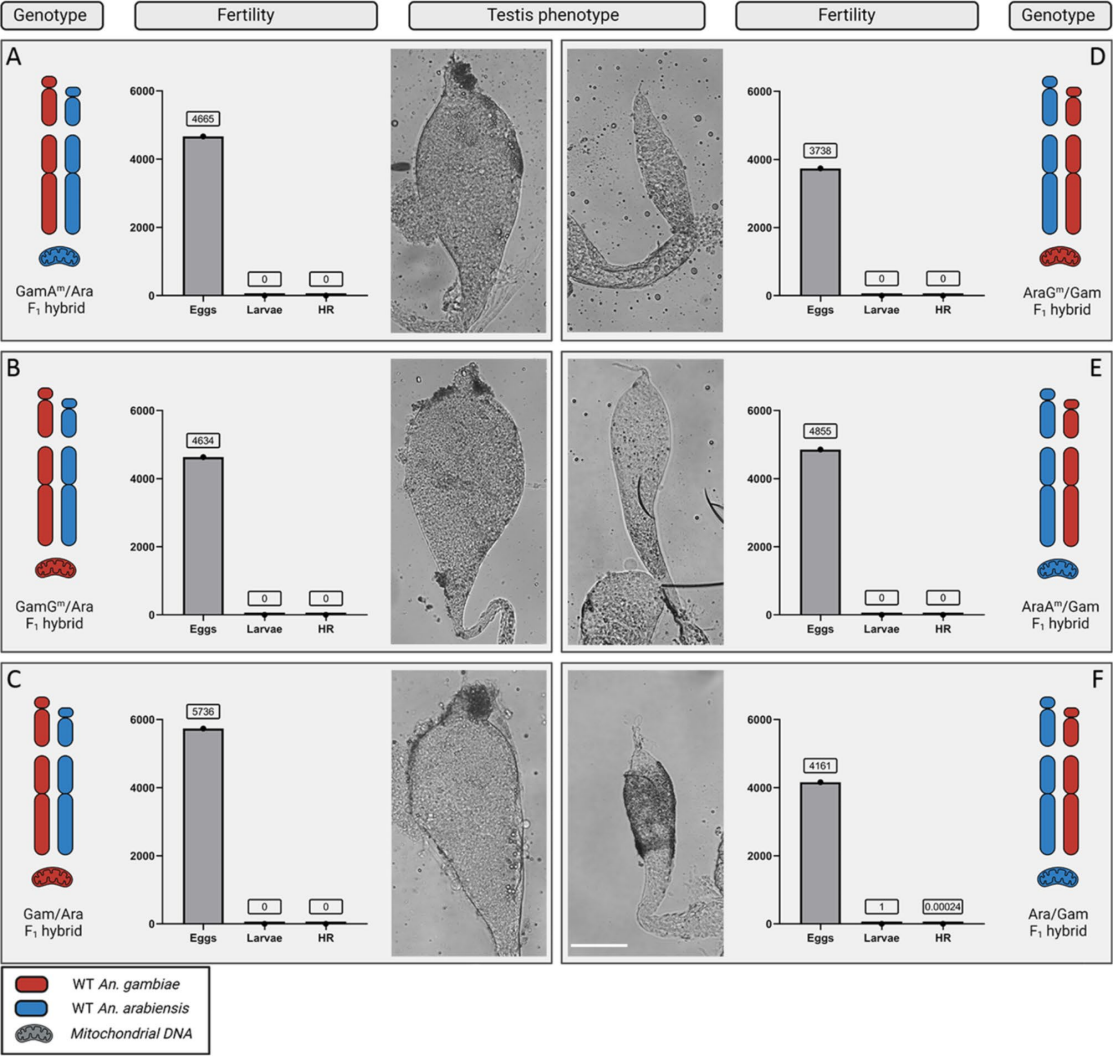
Result of fertility assays and analysis of testes phenotypes associated with F_1_ hybrids with different genetic background. Representative chromosomes are labelled with X (X chromosome), Y (Y chromosome) and A (autosome). Red indicates *An. gambiae* DNA, blue indicates *An. arabiensis* DNA. Testes were dissected at pupal stage for all the males analysed. (**A**, ***B***) and **C** show the outcome of the genetic crosses described in Fig. 3. All cohorts of F_1_ hybrid males that were analysed showed full sterility (no eggs hatching) and the cytological phenotype of their testes was classified as ‘marbled’ with no evident differences to the control samples (**C**). **D**, **E** and **F** show the outcome of the crosses described in Fig. 4. AraG^m^/Gam and AraA^m^/Gam F_1_ hybrid males (**D** and **E**) showed full sterility (no eggs hatching) while from the cross of Ara/Gam F_1_ hybrid males (**F**) a single larva hatched, likely due to experimental error. The cytological phenotype of the testes in these male cohorts showed no evident differences relative to the control samples (*F*). Scale bar 100 μm.

A total of 4665 and 3738 eggs were recovered from the experimental cages of the GamA^m^ and AraG^m^ strains, respectively. No hatching of eggs was observed in either case, indicating the presence of full sterility in these males regardless of the mitochondrial genomes (Fig. 5A and D). In addition, testes in these hybrids displayed the phenotypes expected based on the species identity of the X chromosome. As such, hybrid males carrying the *An. gambiae* X chromosome displayed the marbled phenotype, while those carrying the *An. arabiensis* X chromosome were found to contain atrophic testes. Testes phenotypes, therefore, did not differ between the cohorts of introgressed-strain hybrids and the respective WT control hybrid males (Figs. 1 and 5B, C, E and F).

Notably, a single larvae hatched from the cross of WT F_1_ hybrid males containing *An. arabiensis* X chromosomes (4161 eggs; HR: 0.00024) (Fig. 5F). The full sterility of F_1_ hybrid males with this genetic composition has been well-documented and established in countless experiments within our lab. We cannot rule out that this event was the result of contamination.

### Verifying the species-of-origin of the mitochondrial DNA

The mitochondrial introgressions in this study relied on repeated backcrosses of hybrid female mosquitoes to *An. gambiae* WT males. We wanted to exclude the possibility that errors had occurred over the course of the introgression that may have contaminated the mitochondrial DNA content in the cohorts of hybrid males.

Mitochondrial DNA sequencing data, obtained via whole genome sequencing, was analysed from individual and pooled *An. gambiae* G3 and *An. arabiensis* Dongola strain mosquitoes from our laboratory colonies. A total of 5 single nucleotide polymorphisms (SNPs) were identified that were fixed within the respective strains and could serve as markers to identify the species-of-origin of the mitochondria (Table 1).

**Table 1:** List of fixed strain-specific SNPs in the mitochondrial genome of *an. Gambiae* G3 and *An. Arabiensis* Dongola strains.

Chromosome Coordinates	AgamP4 Reference Allele	Fixed *An. gambiae* G3 Allele	Fixed *An. arabiensis *Dongola Allele
**7255**	A	A	T
**7654**	A	A	G
12,168	C	C	T
15,189	A	A	G
15,243	T	T	C

This table lists all the loci in the mitochondrial genome at which fixed, strain-specific SNPs were identified. The second and third columns indicate the alleles that were present at the marker loci in the AgamP4 reference genome and in the analysed *An. gambiae* G3 strain samples, respectively. The fourth column indicates the alleles that were present at the marker loci in samples of the *An. arabiensis* Dongola strain. The coordinates in bold represent the locations of the SNPs used in this study.

DNA was extracted from different cohorts of hybrid males (6 GamA^m^ /Ara, 8 GamG^m^ /Ara, 8 Gam/Ara, 8 AraG^m^/Gam, 10 AraA^m^/Gam and 10 Ara/Gam F_1_ hybrid males) and a region encompassing two of the SNP markers (at positions 7255 and 7654) was selected for PCR amplification and Sanger sequencing (Supplementary File 1). The investigation into the genotypes of the samples at the marker positions revealed a pattern for the mitochondrial DNA in line with the expectation, ruling out the possibility that contamination had taken place over the course of the introgression experiments.

## Discussion

This study aimed to explore the role of mitochondrial DNA in hybrid incompatibility between *An. gambiae* and *An. arabiensis*.

It is important to note that the *An. gambiae* strain (G3) employed in these experiments is a hybrid of *An. gambiae* and *Anopheles coluzzii*. However, these two species are the most recently diverged in the *Anopheles gambiae* complex and the only exception to Haldane’s rule as they produce fertile hybrids of both sexes^56,57^. Therefore, it was not anticipated that this strain’s genetic background would compromise the study’s primary objective (assessing rescue of fertility in F_1_ hybrid males through a switch in the mitochondrial DNA).

In addition, our investigation focused on F_1_ hybrid males. In this genetic background, hybrid incompatibilities lead to full sterility. Any fertility restoration would have been evident through the production of offspring in crosses involving large numbers of males. On the other hand, assessing the impact on fertility in F_1_ hybrid females, which exhibit varying fertility levels, would have been challenging. Nevertheless, this aspect could be explored in future research.

The generation of F_1_ hybrid males between *An. gambiae* and *An. arabiensis* confirmed Haldane’s rule and full sterility^19^. However, cytological defects in their testes vary depending on the directionality of the parental cross. F_1_ hybrid males resulting from a cross of *An. gambiae* females to *An. arabiensis* males exhibit testes with a marbled phenotype. The reciprocal parental cross produces F_1_ hybrid males whose testes are atrophic^16,17,55^. While these findings have previously been described, we show that the atrophic testes phenotype of F_1_ hybrid males does not manifest until males reach adulthood. Notably, at the pupal stage, the testes of these hybrids clearly show the presence of meiotic chromosomes and sperm, which can develop despite DNA condensation defects. These observations suggest the involvement of genetic elements that operate late in development, potentially leading to the degeneration of the reproductive organs.

Regardless of the parental cross, F_1_ hybrid offspring inherit interspecific autosomes, one set from each parental species. This excludes interautosomal incompatibilities from being responsible for the difference in the testis’s phenotype. In addition, previous research has shown that the Y chromosome functions interchangeably between *An. gambiae* and *An. Arabiensis*, thus indicating that it does not harbour loci contributing to hybrid incompatibility^15^. F_1_ hybrid males produced from a cross of *An. gambiae* females to *An. arabiensis* males inherit the X chromosome from *An. gambiae* while F_1_ hybrid males produced from a cross of *An. arabiensis* females to *An. gambiae* males inherit the X chromosome from *An. arabiensis*. For this reason, the differences in the testes of these hybrid males have been attributed to X chromosome-linked genetic elements^14,15^. However, no specific factors involved in HIs have been identified so far.

In addition to the X chromosome, the mitochondria are also maternally inherited by F_1_ hybrid males and thus differ based on the directionality of the parental cross. Hanemaaijer et al. conducted a study where the complete mitochondrial sequences of 70 individual field-collected mosquito specimens were analysed in an attempt to identify species-specific genetic markers for *An. arabiensis*, *An. coluzzii* and *An. gambiae*. None of the identified SNPs were suitable as molecular markers to distinguish these species. The authors suggested that there is no divergent selection in the mitogenome, which episodic hybridizations between these species could explain^54^.

Recombination of mtDNA has been reported in several taxa, including plants, fungi, animals and humans^58–65^. For it to occur, more than one mitochondrial lineage must be present within a cell simultaneously. Due to the maternal inheritance of mtDNA, cells are generally homoplasmic^59^. However, paternal leakage of mtDNA from sperm has been demonstrated in several species^66–69^ and could occasionally cause heteroplasmy, providing the required conditions for recombination events to occur. Sperm contain mitochondria, and it is thought that up to 100 functional mitochondria (and their genomes) enter the oocyte cytoplasm at fertilisation in mammals^70^. In several mammalian species (including mice, cattle and humans), it has been shown that the mitochondria are tagged with ubiquitin, which enables recognition by oocyte proteolytic enzymes and commits sperm-derived mitochondria to destruction before or during the third proteolytic cleavage^70^. Rokas et al. theorised that survival of paternal mitochondria may be higher in the case of interspecific crosses than in within-species mating because molecular recognition systems may be less efficient in such genetic backgrounds^71^. Studies have demonstrated that there is a higher probability of paternal leakage in interspecific relative to intraspecific genetic backgrounds in mice^72^.

To our knowledge, these phenomena have never been investigated in *Anopheles* mosquitoes. In addition, although studies on the role of mtDNA in HIs have been performed using DNA sequencing data, no experimental studies on the matter have been conducted in these mosquito species.

We analysed the mtDNA derived from the laboratory mosquito colonies used in this study. Six SNPs were identified as different between and fixed within each strain. To investigate the potential role of mDNA in HIs, genetic crosses were used to introgress mitochondria from *An. gambiae* into *An. arabiensis* and vice versa. The species-specific markers identified were used to verify that introgression of interspecific mitochondria had been successful. These introgressed strains enabled us to generate F_1_ hybrid males in which the X chromosome and the mitochondria had originated from different species. Fertility assays and an investigation of testes phenotypes were performed to identify potential changes in the full sterility expected in these hybrids due to the presence of the interspecific mDNA and X chromosomes. No obvious fertility defect was detected in any of the experimental crosses, and the testes’ phenotype of F_1_hybrid males did not differ from that of their controls. F_1_ hybrid males with the X chromosome from *An. gambiae* and mitochondria from *An. arabiensis* showed testes with a marbled phenotype. Conversely, F_1_ hybrid males with the X chromosome from *An. arabiensis* and mitochondria from *An. gambiae* showed atrophic testes. These findings strongly suggest that mitochondrial DNA is unlikely to cause the observed sterility in F_1_ hybrid males between the species used in this study. Additionally, it does not appear responsible for the asymmetry in the testes’ phenotype.

The strain-specific SNPs used to monitor the genetic introgression aligned with the maternal inheritance of mitogenomes, confirming this pattern in these mosquito species and suggesting no mitochondrial recombination had occurred.

It is important to underline that these experiments were conducted in mosquito laboratory colonies, with a primary focus on the role of mtDNA in the context of post-zygotic His. For example, the impact of sexual selection was not explored. As a result, these findings may not entirely reflect what occurs in natural populations. It is possible that the presence of mitochondria within an interspecific genome may impose fitness costs in natural environments, which may not have been observed in the laboratory setting.

Finally, the recent report on a unique mtDNA lineage found in field-caught *An. Coluzzii* has brought attention to mtDNA variation within the *Anopheles gambiae* complex and the potential for complex coevolution between mitochondrial and nuclear DNA^73^. This presents an opportunity to expand studies similar to those outlined in this work to accommodate specific genetic backgrounds.

## Materials and methods

### Mosquitoes’ rearing

Mosquitoes were reared under standard conditions in an insectary at 28 °C and 80% relative humidity. Adult mosquitoes were housed in BugDorm-4M1515 insect-rearing cages (W17.5 x D17.5 x H17.5 cm), with standard cage crosses consisting of 50 virgin females and 50 virgin males sexed at the pupal stage. Larger crosses, consisting of more than 100 individuals, were instead housed in BugDorm-4M2222 insect rearing cages (W24.5 x D24.5 x H24.5 cm). For food, a 5% w/v glucose solution was added to a 25 ml glass bottle, and a Whatman grade 1 filter paper sheet was rolled up and partly submerged in the solution. The adult mosquitoes could land on the filter paper and feed on the glucose solution. Adult males and females were allowed to mate for approximately 6–7 days following emergence, at which point females were allowed to feed on bovine blood using a Hemotek membrane feeding system. Three days after the blood meal, egg bowls were added to the cage to allow females to lay eggs. Egg bowls consisted of 120 ml plastic cups to which rearing water (dH2O supplemented with 0.1% sodium chloride ) was added. In addition, the sides of each cup were covered with partially submerged filter paper in order to avoid eggs sticking to the plastic cup and drying out. Two days following oviposition, when the larvae had hatched, an average of 200 larvae were transferred to plastic trays (85(H) x 264(W) x 238(D)mm) containing rearing water and fish food pellets (NISHIKOI ™). Larvae were kept in trays until pupation. Pupae were collected and, following selection of a specific sex and/or screening for the presence of a transgenic marker, were placed into cups containing rearing water and allowed to emerge in cages.

### Mosquito strains used for the experiments

The WT strains used in the experiments in this project, obtained from MR4, were the *An. arabiensis* Dongola strain (MRA-856) and the *An. gambiae* G3 strain (MRA-112). The G3 strain was isolated from the Gambia in 1975 and has since been maintained in a laboratory setting by the Malaria Research and Reference Reagent Resource Centre (MR4) (MRA-112). The strain is polymorphic for the 2La inversion. The *An. arabiensis* Dongola strain was collected in Sudan in 2004 and was also obtained from MR4 (MRA-856). As in all *An. arabiensis* strains, the 2La inversion is fixed in this strain. In addition, the strain is polymorphic for inversions 2Ra and 2Rb and has a fixed inversion on chromosome 3R. The X-insertion strain is a transgenic *An. gambiae* G3 strain expressing GFP under the control of the 3xP3 promoter from an insertion site on the X chromosome.

### Hybrid fertility assessment (HFA)

To carry out a Cage HFA experiment, males or females of the introgressed strains were crossed to WT *An. gambiae* or *An. arabiensis* females in a standard cage cross to generate F_1_ progeny. F_1_ hybrid males were selected at the pupal stage (when the sexual dimorphism is apparent) and collected in cages until eclosion. Each group of F_1_ hybrid males was crossed to WT *An. gambiae* females in standard cage crosses. Following 6–7 days of mating, females were administered a blood meal and, two days later, allowed to oviposit in an egg bowl. The clutches were monitored for hatching for 4–6 days following oviposition.

### DNA extraction and polymerase chain reaction (PCR)

According to the manufacturer protocol, genomic DNA was extracted using Qiagen DNeasy^®^ Blood & Tissue Kit. DNA was isolated from pooled (6 individuals) F_1_ hybrids generated from each experimental condition (see Figs. 3 and 4). The region of interest containing the SNP was amplified from the mitogenome using the primers F: CGGTATTCTTATTCTTAAGCTTCCT and R: GCTCCTCCAACGTTAAATTTATTAG. The reaction was performed using Phusion^®^ High-Fidelity PCR Master Mix following the manufacturer protocol. The PCR product was purified using the QIAquick^®^ PCR Purification Kit, and the amplicons were sequenced using Eurofins.

### Bioinformatic pipeline and tools

To identify species-specific mitochondrial SNP markers, whole genome sequencing (WGS) data of 10 pooled samples of 10 WT *An. gambiae* G3 adults was aligned to the AgamP4 reference genome, obtained from Vectorbase (Giraldo-Calderón et al., 2015), and variants were called. Fixed SNPs were identified and incorporated into the AgamP4 reference genome to create a G3 pseudoreference genome. WGS data from 2 individual males and a pooled sample of 38 WT *An. arabiensis* Dongola females was aligned to the G3 pseudoreference genome, variants were called and fixed SNPs for *An. arabiensis* were identified. The DNA sequencing data analysed are available in the NCBI Sequence Read Archive repository (Bioprojects PRJNA381033, PRJNA304755 and PRJNA1127581).

A total of 5 fixed SNP differences were identified between the mitochondrial genomes of *An. arabiensis* Dongola and *An. gambiae* G3 (Table 1). The two markers at positions 15,189 and 15,243 were located in a region of the mitogenome that was excluded from the analysis by Hanemaaijer et al. (2019), these markers were not selected for sequencing as primers design was hindered by the AT-rich nature of the region. Instead, primers were designed to amplify a region of DNA containing the SNP markers at positions 7255 and 7654 (Forward primer: CGGTATTCTTATTCTTAAGCTTCCT; Reverse primer: GCTCCTCCAACGTTAAATTTATTAG). DNA was extracted from 6 GamA^m^/Ara (hybrid males with *An. gambiae* X chromosome and mitochondria from *An. arabiensis*), 8 GamG^m^ /Ara (hybrid males with *An. gambiae* X chromosome and mitochondria from *An. gambiae*), 8 Gam/Ara (control hybrid males with *An. gambiae* X chromosome and mitochondria from *An. gambiae*), 8 AraG^m^/Gam (hybrid males with *An. arabiensis* X chromosome and mitochondria from *An. gambiae*), 10 AraA^m^/Gam (hybrid males with *An. arabiensis* X chromosome and mitochondria from *An. arabiensis*),10 Ara/Gam (control hybrid males with *An. arabiensis* X chromosome and mitochondria from *An. arabiensis*) and used for Sanger sequencing and genotyping using Integrative Genomics Viewer (IGV). Sequencing results are available in Supplementary File 1.

The bioinformatic pipeline used to identify the species-specific SNPs in this study is described in Antonios Kriezis’s thesis available online (10.25560/103207).

### Testes dissection and whole-mount DAPI staining

Testes were dissected from pupal or adult stages from WT or F_1_ hybrid mosquitoes in a drop of fresh 1X PBS. For a quick analysis of the testis’s cytological phenotype (Fig. 5), testes were transferred in a clean drop of 1x PBS, and a coverslip was gently applied to the slide. Pictures of the testes were acquired using the cell imaging system EVOS (AMG) using phase contrast at 20x magnification. For Whole mount DAPI staining, following the dissection, testes were fixed in 3.7% Formaldehyde solution in 1X PBS for 10 min at Room Temperature (RT) and then washed 3 times in 0.1% Tween PBS 1X for 15 min. Testes were then mounted in a clean microscope slide in ProLong™ Gold Antifade Mountant with DAPI (ThermoFisher), and the coverslip was sealed using Cytobond (Scigene). Following at least 2 h of incubation at RT, testes were analysed using a Leica SP8 inverted confocal microscope and a 40x oil immersion objective was used.

## Electronic supplementary material

Below is the link to the electronic supplementary material.

Supplementary Material 1

Supplementary Material 2

## Data Availability

The datasets analysed during the current study are available in the NCBI Sequence Read Archive repository (Bioprojects PRJNA381033, PRJNA304755 and PRJNA1127581).

## References

[1] TEAM, W. *World malaria report 2022*. (2022).

[2] Sinka, M. E. et al. A global map of dominant malaria vectors. *Parasit. Vectors*. **5**, 69 (2012). 22475528 10.1186/1756-3305-5-69 PMC3349467

[3] Barrón, M. G. et al. A new species in the major malaria vector complex sheds light on reticulated species evolution. *Sci. Rep.* **9**, 1–13 (2019). 31611571 10.1038/s41598-019-49065-5 PMC6791875

[4] CHARLWOOD, J. D. & JONES, M. D. R. Mating behaviour in the mosquito, Anopheles gambiae s.1.Save. *Physiol. Entomol.* **4**, 111–120 (1979).

[5] Brogdon, W. G. Measurement of flight tone differentiates among members of the Anopheles gambiae species complex (Diptera: Culicidae). *J. Med. Entomol.* **35**, 681–684 (1998). 9775592 10.1093/jmedent/35.5.681

[6] WEKESA, J. W., BESANSKY, N. J. & BROGDON, W. G., HAWLEY, W. A. & Flight tone of field-collected populations of Anopheles gambiae and An. Arabiensis (Diptera: Culicidae). *Physiol. Entomol.* **23**, 289–294 (1998).

[7] Polerstock, A. R., Eigenbrode, S. D. & Klowden, M. J. Mating alters the Cuticular hydrocarbons of Female Anopheles gambiae Sensu Stricto and Aedes aegypti (Diptera: Culicidae). *J. Med. Entomol.* **39**, 545–552 (2002). 12061454 10.1603/0022-2585-39.3.545

[8] Caputo, B. et al. Identification and composition of cuticular hydrocarbons of the major afrotropical malaria vector Anopheles gambiae s.s. (Diptera: Culicidae): analysis of sexual dimorphism and age-related changes. *J. Mass. Spectrom.* **40**, 1595–1604 (2005). 16320293 10.1002/jms.961

[9] Diabaté, A. et al. Mixed swarms of the molecular M and S forms of Anopheles gambiae (Diptera: Culicidae) in sympatric area from Burkina Faso. *J. Med. Entomol.* **43**, 480–483 (2006). 16739404 10.1603/0022-2585(2006)43[480:msotmm]2.0.co;2

[10] Diabaté, A. et al. Spatial swarm segregation and reproductive isolation between the molecular forms of Anopheles gambiae. *Proc. Biol. Sci.* **276**, 4215–4222 (2009). 19734189 10.1098/rspb.2009.1167 PMC2821344

[11] Pennetier, C., Warren, B., Dabiré, K. R., Russell, I. J. & Gibson, G. Singing on the Wing’ as a mechanism for Species Recognition in the Malarial Mosquito Anopheles gambiae. *Curr. Biol.* **20**, 131–136 (2010). 20045329 10.1016/j.cub.2009.11.040

[12] Dabire, K. R. et al. Assortative mating in mixed swarms of the mosquito Anopheles gambiae s.s. M and S molecular forms, in Burkina Faso, West Africa. *Med. Vet. Entomol.* **27**, 298–312 (2013). 23360106 10.1111/j.1365-2915.2012.01049.x

[13] Davidson, G. Anopheles gambiae Complex. *Nature* **196**, 907 (1962).

[14] Slotman, M., Della Torre, A. & Powell, J. R. The genetics of inviability and male sterility in hybrids between Anopheles gambiae and An. Arabiensis. *Genetics* **167**, 275 (2004). 15166154 10.1534/genetics.167.1.275 PMC1470845

[15] Bernardini, F. et al. Cross-species Y chromosome function between malaria vectors of the Anopheles gambiae species complex. *Genetics* **207**, 729–740 (2017). 28860320 10.1534/genetics.117.300221 PMC5629335

[16] Liang, J. & Sharakhov, I. V. Premeiotic and meiotic failures lead to hybrid male sterility in the Anopheles gambiae complex. *Proc. R. Soc. B Biol. Sci.* 286, (2019). 10.1098/rspb.2019.1080 PMC6650711 31288705

[17] Liang, J., Hodge, J. M. & Sharakhov, I. V. Asymmetric phenotypes of sterile hybrid males from reciprocal crosses between species of the Anopheles gambiae Complex. *Front. Ecol. Evol.* **9**, 375 (2021).

[18] Fontaine, M. C. et al. Extensive introgression in a malaria vector species complex revealed by phylogenomics. *Sci. (80-)*. **347**, 1258524 (2015). 10.1126/science.1258524 PMC4380269 25431491

[19] Cowell, F. 100 years of Haldane’s rule. *J. Evol. Biol.*10.1111/jeb.14112 (2022). 36357993 10.1111/jeb.14112 PMC10098713

[20] Neafsey, D. E. et al. SNP genotyping defines complex gene-flow boundaries among African malaria vector mosquitoes. *Science* **330**, 514–517 (2010). 20966254 10.1126/science.1193036 PMC4811326

[21] Mallet, J., Besansky, N. & Hahn, M. W. How reticulated are species? *BioEssays* 38, 140–149 (2016). 10.1002/bies.201500149 PMC4813508 26709836

[22] Pombi, M. et al. Dissecting functional components of reproductive isolation among closely related sympatric species of the Anopheles gambiae complex. *Evol. Appl.* **10**, 1102–1120 (2017). 29151864 10.1111/eva.12517 PMC5680640

[23] Lee, Y. et al. Spatiotemporal dynamics of gene flow and hybrid fitness between the M and S forms of the malaria mosquito, Anopheles gambiae. *Proc. Natl. Acad. Sci.* 110, 19854–19859 (2013). 10.1073/pnas.1316851110 PMC3856788 24248386

[24] Temu, E. A., Hunt, R. H., Coetzee, M., Minjas, J. N. & Shiff, C. J. Detection of hybrids in natural populations of the Anopheles gambiae complex by the rDNA-based, PCR method. *Ann. Trop. Med. Parasitol.* **91**, 963–965 (1997). 9579219 10.1080/00034989760383

[25] Toure, Y. T. et al. The distribution and inversion polymorphism of chromosomally recognized taxa of the Anopheles gambiae complex in Mali, West Africa. *Parassitologia* **40**, 477–511 (1998). 10645562

[26] Tripet, F. et al. DNA analysis of transferred sperm reveals significant levels of gene flow between molecular forms of Anopheles gambiae. *Mol. Ecol.* **10**, 1725–1732 (2001). 11472539 10.1046/j.0962-1083.2001.01301.x

[27] Mawejje, H. D. et al. Insecticide resistance monitoring of field-collected anopheles gambiae s.l. populations from Jinja, eastern Uganda, identifies high levels of pyrethroid resistance. *Med. Vet. Entomol.* **27**, 276–283 (2013). 23046446 10.1111/j.1365-2915.2012.01055.x PMC3543752

[28] Zouré, A. A. et al. Genetic analysis and population structure of the Anopheles gambiae complex from different ecological zones of Burkina Faso. *Infect. Genet. Evol. J. Mol. Epidemiol. Evol. Genet. Infect. Dis.* **81**, 104261 (2020). 10.1016/j.meegid.2020.104261 32092481

[29] Lanzaro, G. C. & Lee, Y. Speciation in Anopheles gambiae — The Distribution of Genetic Polymorphism and Patterns of Reproductive Isolation Among Natural Populations. in (ed. Manguin, S.) Ch. 6IntechOpen, (2013). 10.5772/56232

[30] Dobzhansky, T. Studies on hybrid sterility. II. Localization of sterility factors in Drosophila Pseudoobscura hybrids. *Genetics* **21**, 113–135 (1936). 17246786 10.1093/genetics/21.2.113 PMC1208664

[31] Coyne, J. A. & Charlesworth, B. Location of an X-linked factor causing sterility in male hybrids of Drosophila simulans and D. Mauritiana. *Heredity (Edinb)*. **57** (Pt 2), 243–246 (1986). 3781872 10.1038/hdy.1986.114

[32] Cabot, E. L., Davis, A. W., Johnson, N. A. & Wu, C. I. Genetics of reproductive isolation in the Drosophila simulans clade: complex epistasis underlying hybrid male sterility. *Genetics* **137**, 175–189 (1994). 8056308 10.1093/genetics/137.1.175 PMC1205934

[33] Coyne, J. A. & Orr, H. A. *Speciation* (Sinauer Associates, 2004).

[34] Wu, C. I. & Ting, C. T. Genes and speciation. *Nat. Rev. Genet.* **5**, 114–122 (2004). 14735122 10.1038/nrg1269

[35] Brideau, N. J. et al. Two Dobzhansky-Muller genes interact to cause hybrid lethality in Drosophila. *Science* **314**, 1292–1295 (2006). 17124320 10.1126/science.1133953

[36] Sweigart, A. L., Fishman, L. & Willis, J. H. A simple genetic incompatibility causes Hybrid Male sterility in Mimulus. *Genetics* **172**, 2465–2479 (2006). 16415357 10.1534/genetics.105.053686 PMC1456371

[37] Masly, J. P. & Presgraves, D. C. High-resolution genome-wide dissection of the two rules of speciation in Drosophila. *PLOS Biol.* **5**, e243 (2007). 17850182 10.1371/journal.pbio.0050243 PMC1971125

[38] Deitz, K. C., Takken, W. & Slotman, M. A. The Genetic Architecture of Post-zygotic Reproductive isolation between Anopheles coluzzii and an. Quadriannulatus. *Front. Genet.* **11**, p.925 (2020). 10.3389/fgene.2020.00925 PMC7480394 33005168

[39] della Torre, A., Merzagora, L., Powell, J. R. & Coluzzi, M. Selective introgression of paracentric inversions between two sibling species of the Anopheles gambiae complex. *Genetics* **146**, 239–244 (1997). 9136013 10.1093/genetics/146.1.239 PMC1207938

[40] Slotman, M. A., Della Torre, A., Calzetta, M. & Powell, J. R. Differential introgression of chromsomal regions between Anopheles gambiae and An. Arabiensis. *Am. J. Trop. Med. Hyg.* **73**, 326–335 (2005). 16103599

[41] Burton, R. S., Pereira, R. J. & Barreto, F. S. Cytonuclear genomic interactions and hybrid breakdown. *Annu. Rev. Ecol. Evol. Syst.* **44**, 281–302 (2013).

[42] Gershoni, M., Templeton, A. R. & Mishmar, D. Mitochondrial bioenergetics as a major motive force of speciation. *Bioessays* **31**, 642–650 (2009). 19408245 10.1002/bies.200800139

[43] Ma, H. et al. Incompatibility between Nuclear and mitochondrial genomes contributes to an Interspecies Reproductive Barrier. *Cell. Metab.* **24**, 283–294 (2016). 27425585 10.1016/j.cmet.2016.06.012 PMC4981548

[44] Cuperfain, A. B., Zhang, Z. L., Kennedy, J. L. & Gonçalves, V. F. The Complex Interaction of Mitochondrial Genetics and mitochondrial pathways in Psychiatric Disease. *Mol. Neuropsychiatry*. **4**, 52–69 (2018). 29998118 10.1159/000488031 PMC6032034

[45] Osellame, L. D., Blacker, T. S. & Duchen, M. R. Cellular and molecular mechanisms of mitochondrial function. *Best Pract. Res. Clin. Endocrinol. Metab.* **26**, 711–723 (2012). 23168274 10.1016/j.beem.2012.05.003 PMC3513836

[46] Sackton, T. B., Haney, R. A. & Rand, D. M. Cytonuclear coadaptation in Drosophila: disruption of cytochrome c oxidase activity in backcross genotypes. *Evolution* **57**, 2315–2325 (2003). 14628919 10.1111/j.0014-3820.2003.tb00243.x

[47] Keaney, T. A., Wong, H. W. S., Dowling, D. K., Jones, T. M. & Holman, L. Mother’s curse and indirect genetic effects: do males matter to mitochondrial genome evolution? *J. Evol. Biol.* **33**, 189–201 (2020). 31650630 10.1111/jeb.13561

[48] Ålund, M. et al. Tracking hybrid viability across life stages in a natural avian contact zone. *Evol. (N Y)*. **78**, 267–283 (2024). 10.1093/evolut/qpad204 37952134

[49] Munasinghe, M., Haller, B. C. & Clark, A. G. Migration restores hybrid incompatibility driven by mitochondrial–nuclear sexual conflict. *Proc. R. Soc. B Biol. Sci.* 289, 20212561 (2022). 10.1098/rspb.2021.2561 PMC8790342 35078356

[50] DAVIDSON, G. Anopheles gambiae, a complex of species. *Bull. World Health Organ.* **31**, 625 (1964). 14278001 PMC2555133

[51] Davidson, G. & The five mating-types in the anopheles gambiae complex. *Riv Malariol* **43**, 167-183 (1964). 14318975

[52] Lynch, M., Koskella, B. & Schaack, S. Mutation pressure and the evolution of organelle genomic architecture. *Science* **311**, 1727–1730 (2006). 16556832 10.1126/science.1118884

[53] Allio, R., Donega, S., Galtier, N. & Nabholz, B. Large variation in the ratio of mitochondrial to Nuclear Mutation Rate across animals: implications for genetic diversity and the Use of mitochondrial DNA as a molecular marker. *Mol. Biol. Evol.* **34**, 2762–2772 (2017). 28981721 10.1093/molbev/msx197

[54] Hanemaaijer, M. J. et al. Mitochondrial genomes of Anopheles arabiensis, an. Gambiae and An. Coluzzii show no clear species division. *F1000Research* **7**, 1–14 (2019). 10.12688/f1000research.13807.1 PMC6489993 31069048

[55] Bernardini, F., Kriezis, A., Galizi, R., Nolan, T. & Crisanti, A. Introgression of a synthetic sex ratio distortion system from Anopheles gambiae into Anopheles arabiensis. *Sci. Rep. 2019*. **91 9**, 1–8 (2019). 10.1038/s41598-019-41646-8 PMC6435806 30914785

[56] Aboagye-Antwi, F. et al. Experimental swap of Anopheles gambiae’s assortative mating preferences demonstrates key role of X-Chromosome Divergence Island in Incipient Sympatric Speciation. *PLOS Genet.* **11**, e1005141 (2015). 25880677 10.1371/journal.pgen.1005141 PMC4400153

[57] Diabaté, A., Dabire, R. K., Millogo, N. & Lehmann, T. Evaluating the effect of postmating isolation between Molecular forms of Anopheles gambiae (Diptera: Culicidae). *J. Med. Entomol.* **44**, 60–64 (2007). 17294921 10.1603/0022-2585(2007)44[60:eteopi]2.0.co;2

[58] Lunt, D. H. & Hyman, B. C. Animal mitochondrial DNA recombination. *Nature* **387**, 247 (1997). 9153388 10.1038/387247a0

[59] Birky, C. W. The inheritance of genes in Mitochondria and chloroplasts: laws, mechanisms, and models. *Annu. Rev. Genet.* **35**, 125–148 (2001). 11700280 10.1146/annurev.genet.35.102401.090231

[60] Ladoukakis, E. D. & Zouros, E. Recombination in animal mitochondrial DNA: evidence from published sequences. *Mol. Biol. Evol.* **18**, 2127–2131 (2001). 11606710 10.1093/oxfordjournals.molbev.a003755

[61] Hoarau, G., Holla, S., Lescasse, R., Stam, W. T. & Olsen, J. L. Heteroplasmy and evidence for recombination in the mitochondrial Control Region of the Flatfish Platichthys flesus. *Mol. Biol. Evol.* **19**, 2261–2264 (2002). 12446816 10.1093/oxfordjournals.molbev.a004049

[62] Kraytsberg, Y. et al. Recombination of human mitochondrial DNA. *Science* **304**, 981 (2004). 15143273 10.1126/science.1096342

[63] Gantenbein, B., Fet, V., Gantenbein-Ritter, I. A. & Balloux, F. Evidence for recombination in scorpion mitochondrial DNA (Scorpiones: Buthidae). *Proceedings. Biol. Sci.* 272, 697–704 (2005). 10.1098/rspb.2004.3017 PMC1602043 15870032

[64] Piganeau, G., Gardner, M. & Eyre-Walker, A. A. Broad survey of recombination in Animal Mitochondria. *Mol. Biol. Evol.* **21**, 2319–2325 (2004). 15342796 10.1093/molbev/msh244

[65] Ciborowski, K. L. et al. Rare and fleeting: an example of interspecific recombination in animal mitochondrial DNA. *Biol. Lett.* **3**, 554–557 (2007). 17650476 10.1098/rsbl.2007.0290 PMC2396188

[66] Gyllensten, U., Wharton, D., Josefsson, A. & Wilson, A. C. Paternal inheritance of mitochondrial DNA in mice. *Nature* **352**, 255–257 (1991). 1857422 10.1038/352255a0

[67] Magoulas, A. & Zouros, E. Restriction-site heteroplasmy in Anchovy (Engraulis encrasicolus) indicates incidental biparental inheritance of mitochondrial DNA. *Mol. Biol. Evol.* **10**, 319 (1993).

[68] Schwartz, M. & Vissing, J. Paternal inheritance of mitochondrial DNA. *N Engl. J. Med.* **347**, 576–580 (2002). 12192017 10.1056/NEJMoa020350

[69] Kvist, L., Martens, J., Nazarenko, A. A. & Orell, M. Paternal leakage of mitochondrial DNA in the great tit (Parus major). *Mol. Biol. Evol.* **20**, 243–247 (2003). 12598691 10.1093/molbev/msg025

[70] Sutovsky, P. et al. Ubiquitinated sperm mitochondria, selective proteolysis, and the regulation of mitochondrial inheritance in mammalian embryos. *Biol. Reprod.* **63**, 582–590 (2000). 10906068 10.1095/biolreprod63.2.582

[71] Rokas, A., Ladoukakis, E. & Zouros, E. Animal mitochondrial DNA recombination revisited. *Trends Ecol. Evol.* **18**, 411–417 (2003).

[72] Shitara, H., Hayashi, J. I., Takahama, S., Kaneda, H. & Yonekawa, H. Maternal inheritance of mouse mtDNA in interspecific hybrids: segregation of the leaked paternal mtDNA followed by the prevention of subsequent paternal leakage. *Genetics* **148**, 851–857 (1998). 9504930 10.1093/genetics/148.2.851 PMC1459812

[73] Amaya Romero, J. E. et al. Mitochondrial variation in Anopheles gambiae and Anopheles coluzzii: Phylogeographic Legacy and Mitonuclear associations with Metabolic Resistance to pathogens and insecticides. *Genome Biol. Evol.* **16**(9), p.evae172 (2024). 10.1093/gbe/evae172 PMC11370803 39226386

