# Supplementary material for: Unravelling the role of mitochondrial DNA in hybrid incompatibility within species of the Anopheles gambiae complex
Source: Sci Rep. 2024 Nov 27;14:29467. doi: 10.1038/s41598-024-80887-0 (PMC11603187; doi:10.1038/s41598-024-80887-0)
Supplement: Supplementary file 2 — Supplementary Material 2 [file 41598_2024_80887_MOESM2_ESM.pdf]

Unravelling the role of mitochondrial DNA in hybrid incompatibility within species of the *Anopheles* complex.

Antonios Kriezis†, Matteo Vitale†, Giulia Morselli†, Andrea Crisanti†, Federica Bernardini†.

<u>Supplementary Figures.</u>

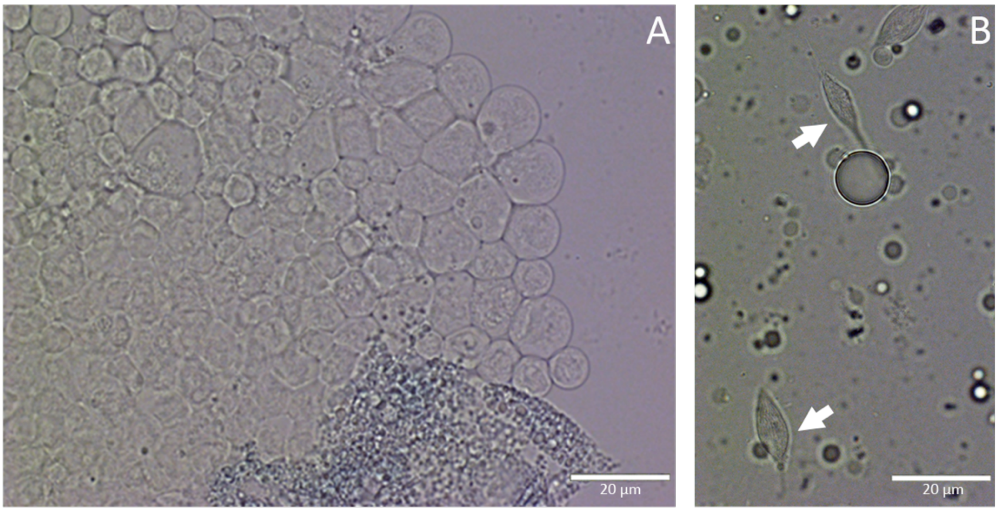

Figure S1: Squashing performed on testis dissected from F_1 hybrid pupae obtained by crossing WT *An. gambiae* females with WT *An. arabiensis* males. (A) Squashing of the testis dissected from F_1 hybrid pupae revealed the presence of large, round, underdeveloped cells. In addition, enlarged spindle-like cells with a partial tail were identified (B). These cells are classified as immature or defective spermatozoa. No motile cells were detected after squashing of the testis.

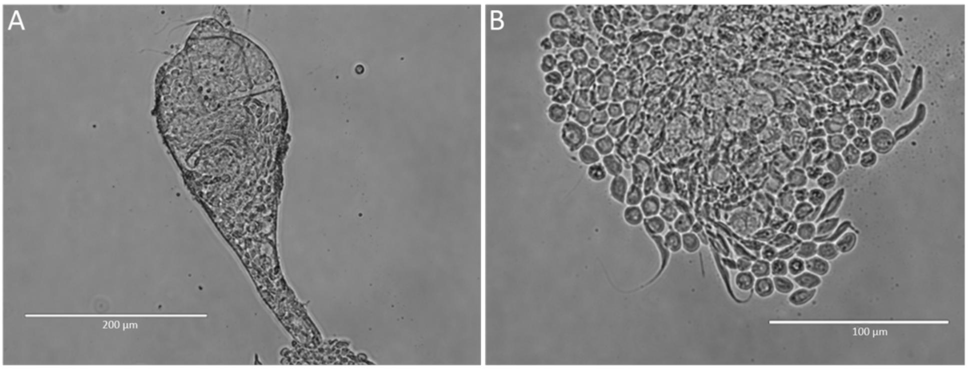

Figure S2: Squashing performed on testes dissected from F_1 hybrid pupae obtained by crossing WT *An. arabiensis* females with *An. gambiae* males. (A) Testis dissected from F_1 hybrid pupae, elongated cells with a partial tail were detected after squashing (B). These sperm-like cells were not motile.

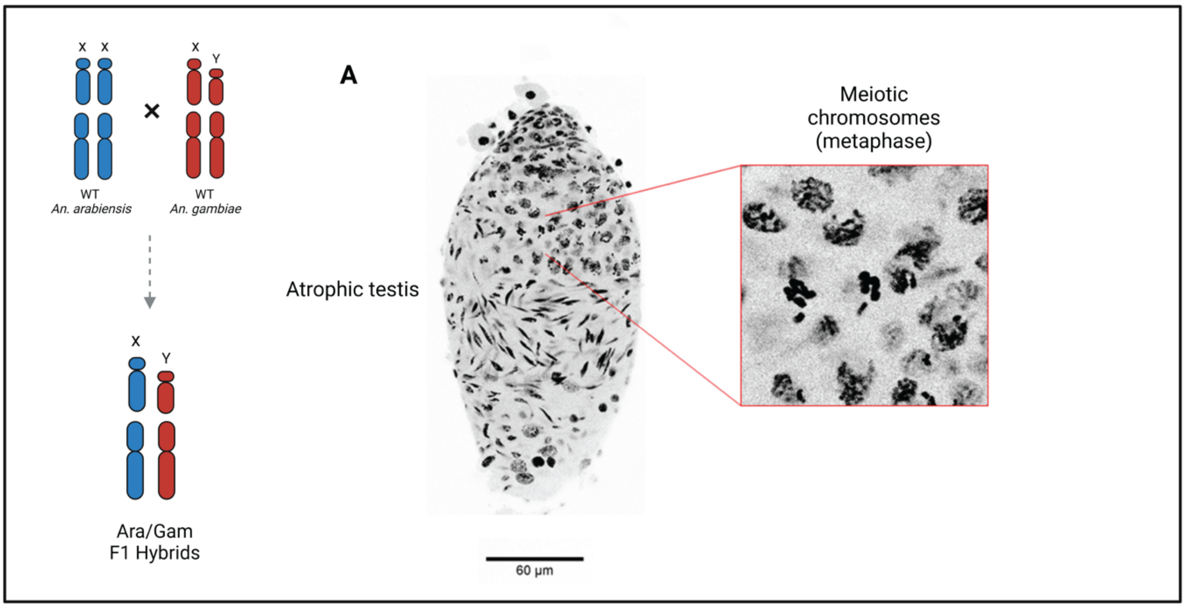

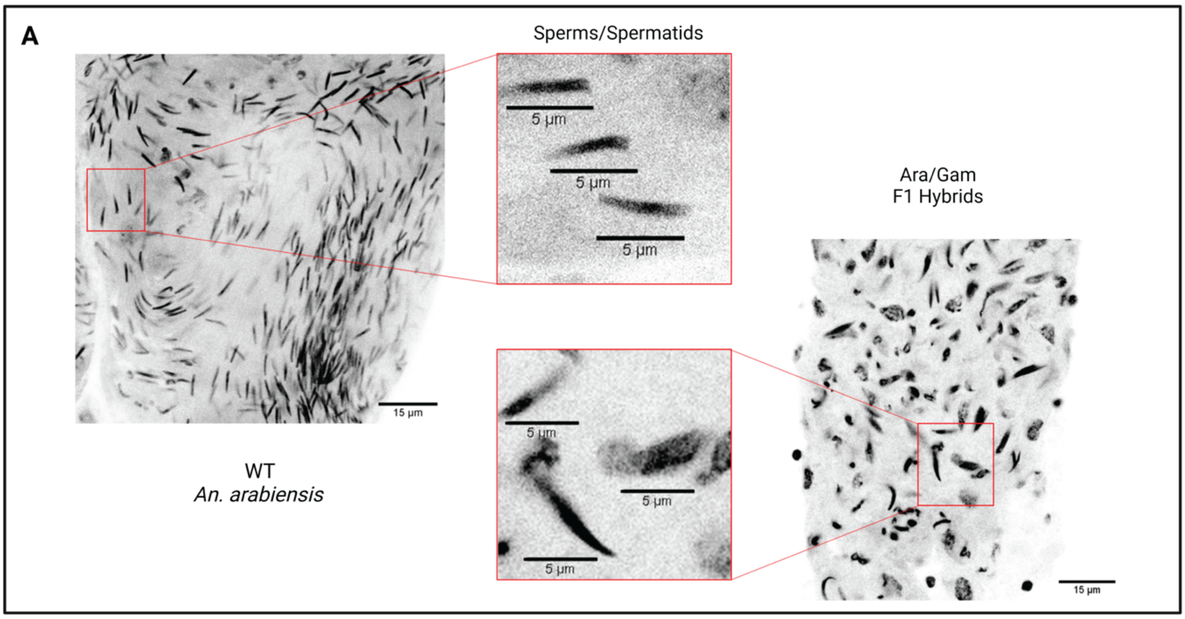

Figure S3: Atrophic testes phenotype in F_1 hybrid males. (A) Testes dissected at pupal stage from Ara/Gam F_1 hybrid males showed the presence of different stages of spermatogenesis. Despite these males are fully sterile, meiotic chromosome and chiasmata are present in the pupal testes. (B) Sperms present in the testes dissected from adult Ara/Gam F_1 hybrid males showed chromatin condensation defects. These sperms are larger and show a high degree of shape variability when compared to sperms in WT *An. arabiensis* males.
